# Experimental Study on Calcination of Portland Cement Clinker Using Different Contents of Stainless Steel Slag

**DOI:** 10.3390/ma17102305

**Published:** 2024-05-13

**Authors:** Jiantao Ju, Haibo Cao, Wenke Guo, Ning Luo, Qiming Zhang, Yonggang Wang

**Affiliations:** 1 School of Metallurgical Engineering, Xi’an University of Architecture and Technology, Xi’an 710200, China; 15309216845@163.com (H.C.); guowwenke@163.com (W.G.); 15795082077@163.com (N.L.); zqm980513@163.com (Q.Z.); 2 Gansu Jiugang Group Hongxing Iron and Steel Co., Ltd., Jiayuguan 735100, China; wangyonggang@jiugang.com

**Keywords:** stainless steel slag, calcination, silicate cement clinker, microstructure, compressive strength

## Abstract

In order to increase the utilization rate of stainless steel slag, reduce storage needs, and mitigate environmental impacts, this study replaces a portion of limestone with varying amounts of stainless steel slag in the calcination of Portland cement clinker. The study primarily examines the influence of stainless steel slag on the phase composition, microstructure, compressive strength, and free calcium oxide (ƒ-CaO) content of Portland cement clinker. The results show the following: (1) Using stainless steel slag to calcine Portland cement clinker can lower the calcination temperature, reducing industrial production costs and energy consumption. (2) With an increase in the amount of stainless steel slag, the dicalcium silicate (C_2_S) and tricalcium silicate (C_3_S) phases in Portland cement clinker initially increase and then decrease; the C_3_S crystals gradually transform into continuous hexagonal plate-shaped distributions, while the tricalcium aluminate (C_3_A) and tetracalcium aluminoferrite (C_4_AF) crystal structures become denser. When the stainless steel slag content is 15%, the dicalcium silicate and tricalcium silicate phases are at their peak; the C_3_S crystals are continuously distributed with a relatively dense structure, and C_3_A and C_4_AF crystals melt and sinter together, becoming distributed around C_3_S. (3) As stainless steel slag content increases, the compressive strength of Portland cement clinker at 3 days, 7 days, and 28 days increases and then decreases, while ƒ-CaO content decreases and then increases. When the stainless steel slag content is 15%, the compressive strength at 28 days is at its highest, 64.4 MPa, with the lowest ƒ-CaO content, 0.78%. The test results provide a basis for the utilization of stainless steel slag in the calcination of Portland cement clinker.

## 1. Introduction

Stainless steel slag is solid waste generated during the production of stainless steel, with approximately 0.78 tons of slag produced for every 3 tons of crude stainless steel [1]. The global annual emissions of stainless steel slag are close to 20 million tons. Stainless steel slag mainly consists of oxides formed by the oxidation of elements contained in molten iron and scrap steel, impurities introduced by metallic furnace materials, slag-forming agents, and eroded refractory materials. Its chemical composition includes not only calcium, silicon, magnesium, sodium, and potassium but also valuable metals, such as nickel, chromium, and iron. Its main mineral phases include dicalcium silicate (C_2_S) and tricalcium silicate (C_3_S), which are cementitious substances [2], and iron-phase solid solution, similar to silicate cement clinker [3,4]. Some toxic substances, such as Cr^6+^ and Ni^2+^, in stainless steel slag are easily leached out, posing corrosive and toxic risks to animals, plants, and humans. Prolonged exposure to metallic chromium can cause corrosion damage to the skin, digestive tract, lungs, etc., and in severe cases, it can have carcinogenic effects. Large quantities of stainless steel slag are still disposed of in landfills, leading to resource wastage. Over time, Cr^6+^ and Ni^2+^ can gradually leach out, infiltrating underground and polluting soil and water, which can severely damage the ecological environment [5]. Therefore, the comprehensive utilization of stainless steel slag has been a subject of concern [6,7]. China’s harmless and resource-based treatment of stainless steel slag is still in the initial stage, mainly focusing on building materials, preparation of microcrystalline glass, ceramic materials, and other fields. However, related research results are mostly in the experimental stage, with a relatively low utilization rate of resources [1].

Liu Xiaoxuan et al. [8] prepared concrete pavement bricks by using stainless steel slag, suggesting that stainless steel slag can replace 50% of the fine sand to prepare concrete pavement bricks with strength grade above Cc30, exhibiting excellent mechanical properties and frost resistance. Dai Jian et al. [9] replaced cement with stainless steel slag and found that with the increase in stainless steel slag content, the strength of cement mortar gradually decreased. Sheen et al. [10] studied the application of stainless steel slag in self-compacting concrete, where 30% stainless steel slag can replace cement, and the compressive strength of self-compacting concrete meets requirements, contributing to cost reduction and environmental protection. Mudersbach et al. [11], Kûhn et al. [12], and others treated deposited stainless steel slag by detoxification methods such as high-temperature environments to produce construction aggregates. Although this method can handle some stainless steel slag, its high energy consumption, large investment, and low output limit its widespread application. Tsakiridis et al. [13] used steel slag to produce silicate cement clinker and found that the use of steel slag does not affect the calcination process of silicate cement clinker or the physical and mechanical properties of silicate cement. Xing Yabing et al. [14] studied the effect of stainless steel slag ultrafine powder on the properties and microstructure of silicate cement, observing that the addition of ultrafine powder can significantly reduce the content of calcium hydroxide in cement and make the cement microstructure denser. E. Haustein et al. [15] investigated the effect of sludge ash substitution for cement on the performance of cement mortar, finding that with the increase in substitution, the rate of heat release decreased, and the total heat was also reduced compared with control cement mortar. J. Rosales [16] studied the potential of stainless steel slag waste in manufacturing self-compacting concrete, demonstrating the feasibility of using stainless steel slag as a replacement for limestone filler to manufacture self-compacting concrete and analyzing the mechanical properties and durability of self-compacting concrete, suggesting its possible application as a building material. Chou et al. [17] studied the flame-retardant and sound insulation properties of fiber-reinforced cement boards using stainless steel electric arc furnace reduction slag, optimizing the ratio of cement boards made from electric arc furnace reduction slag and achieving a sound transmission loss of over 30 decibels across the entire frequency range, which is 3–8 decibels higher than similar specifications on the current construction materials market. M.E. Parron-Rubio [18] investigated the development of sustainable concrete using steel industry dust and slag as substitutes, finding that replacing 25% of cement with materials or waste generated from stainless steel manufacturing processes results in approximately 21% and 25% increases in resistance, indicating the feasibility of using steel industry waste to improve the performance of cement or at least match it. These studies indicate that the comprehensive utilization of stainless steel slag mainly focuses on building materials, preparation of microcrystalline glass, ceramic materials, etc. In terms of building materials, the direct blending of stainless steel slag to replace limestone to prepare silicate cement clinker may reduce the physical properties of silicate cement clinker due to the crystal structure formed by the slow cooling of stainless steel slag at 1600 °C and the presence of ƒ-CaO.

The demand for silicate cement is increasing globally [19], yet the production of silicate cement clinker [20,21] remains a high-energy, high-pollution industry that consumes significant mineral resources and energy [22,23]. The decomposition of limestone and the combustion of fuels during clinker production emit large amounts of CO_2_, making the efficient and clean production of silicate cement an urgent issue to address [24,25,26]. Directly incorporating stainless steel slag into clinker poses a risk of late-stage expansion and cracking due to the presence of free calcium oxide. Therefore, it is preferable to use stainless steel slag in the calcination of Portland cement clinker. The studies mentioned above provide research directions for the comprehensive utilization of stainless steel slag and the clean production of silicate cement clinker [22,27,28]. Stainless steel slag can be used as an independent raw-material component to calcine silicate cement clinker, significantly reducing the calcination temperature and holding time. This not only improves the comprehensive utilization of stainless steel slag and addresses its environmental issues but also provides alternative raw materials for the cement industry, promoting the clean production of silicate cement clinker and achieving carbon emission reduction goals.

Currently, there is little research focusing on the use of stainless steel slag as a raw material for calcining in the production of silicate cement. The purpose of this experiment is to utilize stainless steel slag for calcination to produce silicate cement clinker and to determine the optimal ratio of stainless steel slag. Five different proportions of raw-material samples containing stainless steel slag were prepared. By controlling the lime saturation factor (KH), silica modulus (SM or n), and alumina modulus (IM or p) within appropriate ranges, the raw-material ratio of silicate cement clinker was determined. The optimal calcination temperature and holding time were obtained by high-temperature calcination analysis of the easy-burning properties of silicate cement clinker. Subsequently, burning experiments were conducted on the silicate cement clinker. The effects of different contents of stainless steel slag on the phase composition, microstructure changes, compressive strength, and ƒ-CaO content of silicate cement clinker were analyzed through XRD, SEM-EDS, a constant-loading cement compressive testing machine, and the ethylene glycol–ethanol method, thereby determining the appropriate ratio of stainless steel slag.

## 2. Materials and Methods

### 2.1. Raw Materials

The required raw materials for the experiment, including stainless steel slag, basalt, limestone, copper smelting slag, and silica, were all provided by Jiugang Group. Chemical composition analysis of the five raw materials was conducted by using an X-ray (D8/ADVANCE/A25, Bruker Corporation, Berlin, Germany) fluorescence spectrometer, an atomic absorption spectrometer (PinAAcle 900, Shanghai Metash Instruments CO., Ltd., Shanghai, China), and a high-frequency combustion infrared absorption carbon–sulfur analyzer (SDHFCS1000, Sande Science and Technology, Changsha, China).

### 2.2. Ingredient Design

When conducting cement raw-material calculations, the compositions of cement clinker minerals are designed by using three rate values: lime saturation factor (KH), silica modulus (SM), and alumina modulus (IM). The calculation formulas are as follows [29]:

(1) $$KH=\frac{CaO-1.65Al_{2}O_{3}-0.35Fe_{2}O_{3}}{2.8SiO_{2}}$$

The KH value for Portland cement typically ranges from 0.667 to 1.0. In this experiment, the clinker rate value (KH) was set around 0.911.

(2) $$SM=\frac{SiO_{2}}{Al_{2}O_{3}+Fe_{2}O_{3}}$$

In this experiment, various solid waste ingredients were used. To avoid excessive liquid-phase generation and formation of rings in the kiln, a higher silica modulus was chosen. The designed value of SM in this experiment was approximately 2.47.

(3) $$IM=\frac{Al_{2}O_{3}}{Fe_{2}O_{3}}$$

The alumina modulus (IM) for Portland cement typically ranges from 0.8 to 1.7. In ingredient calculation, a lower alumina modulus is preferred. If the IM is too low, the viscosity of the liquid phase decreases, allowing CaO and SiO_2_ to diffuse more quickly in the liquid phase, thereby accelerating the formation of C_3_S. This is why it is preferable to choose a lower aluminum ratio.

### 2.3. Experimental Procedure

Based on different stainless steel slag contents, five groups of experiments were designed: 0% (S-0), 5% (S-5), 10% (S-10), 15% (S-15), and 20% (S-20). They were ground by using a test ball mill, and the ground materials were uniformly mixed. Samples were collected by multi-point sampling and sieved through a 200-mesh (80 μm) sieve, with the residue being less than 5%. The sieved raw materials were mixed according to Table 1 and then compacted into blocks by using a four-column hydraulic press (model *-YL-32-315, Qingdao Power Control Equipment Technology Co., Qingdao, China) with a mold size of 4 cm × 4 cm. They were placed in a high-temperature box-type resistance furnace (model *-SGM8617CE, Xi’an, China) for calcination according to the schedule in Table 2. Clinker was in the form of a cube with dimensions of approximately 5 cm in length and width, and about 0.5 cm in height. After calcination, the clinker was removed and rapidly cooled to room temperature by using forced air. Then, the clinker was ground by using a sample preparation machine, packaged, and labeled (S-0, S-5, S-10, S-15, and S-20). The ƒ-CaO content of S-0, S-5, S-10, S-15, and S-20 was determined separately to analyze their burnability.

After analyzing the burnability of clinker, the optimal calcination temperature and holding time for S-0, S-5, S-10, S-15, and S-20 were determined as shown in Table 3. The clinker samples obtained from calcining S-0, S-5, S-10, S-15, and S-20 according to Table 3 were denoted by M-0, M-5, M-10, M-15, and M-20, respectively. Subsequently, large-scale calcination experiments were conducted according to M-0, M-5, M-10, M-15, and M-20 to further analyze the mineral phase composition, microscopic structural changes, compressive strength, and ƒ-CaO content changes of clinker. This process aimed to determine the appropriate ratio of stainless steel slag.

### 2.4. Characterization Methods

The partially calcined clinker samples (M-0, M-5, M-10, M-15, and M-20) were ground by using a sample preparation machine and sieved through a 200-mesh (80 μm) sieve, and the sieved material was transferred into sample bags for testing. The remaining clinker samples were stored in a curing box.

The clinker samples were analyzed by using an X-ray diffractometer (D8/ADVANCE/A25; Bruker Corporation, Munich, Germany), with a scanning angle range of 10~90° and a scanning rate of 3°/min. Subsequently, Jade software was utilized for phase analysis and semi-quantitative calculation. The microscopic morphology of clinker was examined by using a scanning electron microscope (Gemini SEM 300; Carl Zeiss AG, Oberkochen, Germany), and the elemental composition and distribution were analyzed by using energy-dispersive X-ray spectroscopy (EDS) (EDX-8100, Shimadzu Corporate Management (China) Limited/Shimadzu (Hong Kong) Limited, Hong Kong, China). For elemental content analysis, it was assumed that the samples were composed entirely of five elements: Ca, Si, Al, Fe, and O. The clinker samples were placed on standard compression molds, and their compressive strength was tested by using a constant-loading cement compression testing machine (YAW-300.10B; Zhejiang Xuantian Technology Co., Ltd., Taizhou, China) at different curing ages (3 days, 7 days, and 28 days), with a loading speed of 2.4 kN/s. The compressive strength data were obtained by averaging the compressive strength of three identical test samples. To extract calcium oxide, appropriate solvents, such as glycerol–ethanol solution or ethylene glycol–ethanol solution, were used to form corresponding calcium salts. Then, the generated calcium salts were titrated with benzoic acid standard solution. Based on the consumed standard solution titration degree and volume, the ƒ-CaO content in the samples was calculated.

## 3. Results and Discussion

### 3.1. Discussion of Raw Materials and Raw-Material Ratios

Table 4 shows the oxide composition table of the raw materials. According to Table 4, the main components in Portland cement are four oxides: CaO, SiO_2_, Al_2_O_3_, and Fe_2_O_3_. Among the raw materials, limestone mainly provides CaO, while silica and basalt primarily supply SiO_2_ and Al_2_O_3_. Copper smelting slag mainly provides Fe_2_O_3_ and SiO_2_. Stainless steel slag, which contains high levels of CaO and SiO_2_, along with a small amount of Fe_2_O_3_, can be used as a partial substitute for limestone and other raw materials. By adopting a multi-component material blending technique to reasonably combine these raw materials, it is possible to meet the chemical composition and mineral content requirements for producing ordinary Portland cement clinker.

**Figure 1:**
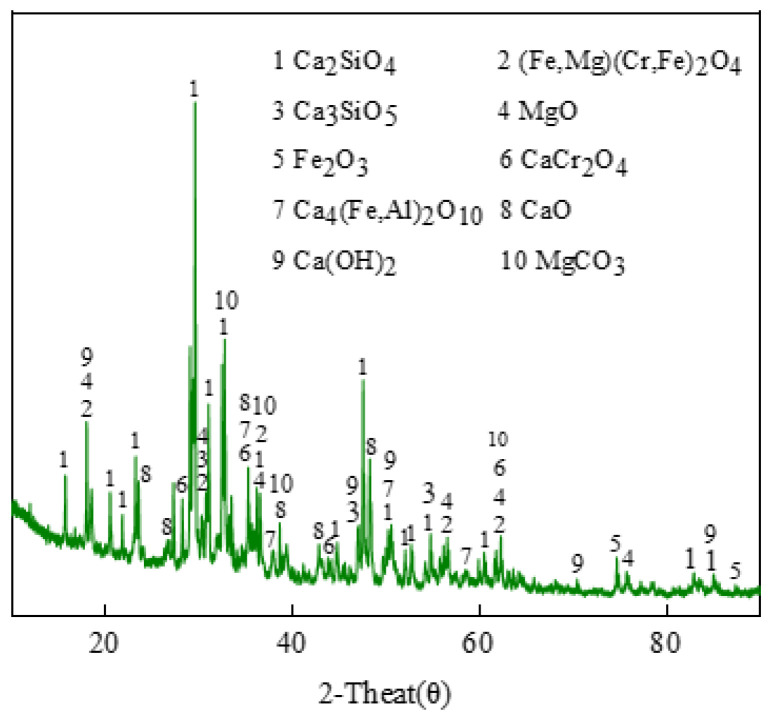
XRD spectrum of stainless steel slag.

Figure 1 shows the XRD analysis results of stainless steel slag, while Table 1 presents the phase contents of stainless steel slag. As seen in Figure 1, the main phases of stainless steel slag were C_2_S and C_3_S, with some calcium forming small amounts of ƒ-CaO and Ca(OH)_2_. Iron was mainly present as hematite (Fe_2_O_3_). According to Table 1, the content of C_2_S in stainless steel slag was 48.0%, and the content of Ca(OH)_2_ was 4.01%. No Cr^6+^ phases were found in stainless steel slag.

For dosage design, according to Section 2.2, the mineral phase composition of cement clinker is calculated based on the selected rate value range. Since a high iron content can reduce the firing temperature of cement, resulting in adverse effects such as false setting and reduced strength, the content of stainless steel slag is taken as the basic variable in the ingredient scheme. The proportions of raw materials for calcining silicate cement clinker are shown in Table 5. Through ingredient calculation, it was inferred that the raw materials consisted of stainless steel slag, basalt, limestone, copper smelting waste slag, and silica. The clinker ratio for KH was around 0.911, the SM value was approximately 2.47, and the IM was generally between 0.8 and 1.7. If the content of stainless steel slag exceeds 20%, the clinker ratio will not meet the desired conditions, indicating that 20% is essentially the upper limit for the proportion of stainless steel slag that can be incorporated into the mix.

### 3.2. Burnability Analysis of Clinker

**Figure 2:**
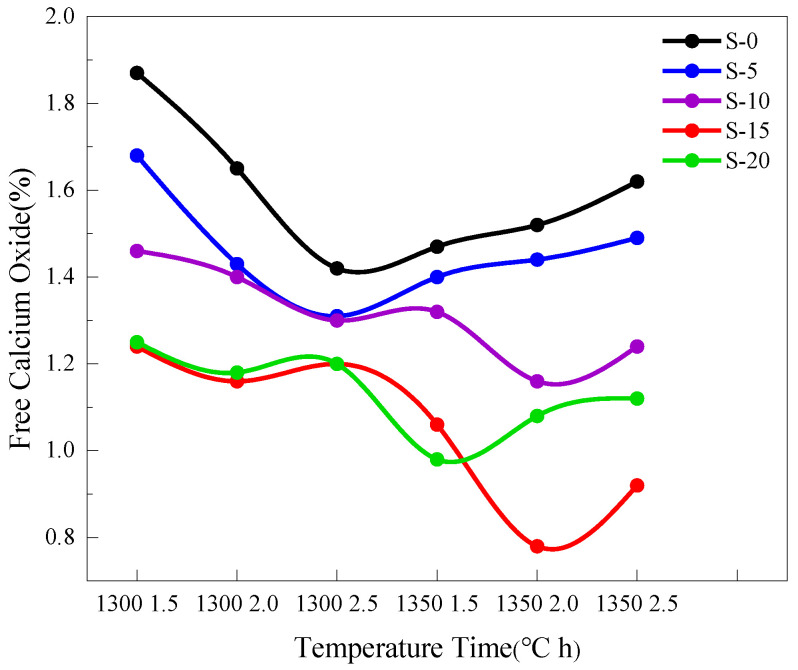
Free calcium oxide contents in S-0, S-5, S-10, S-15, and S-20.

The easy-to-burn test is a fundamental test to assess the ease of cement burning, to determine the burning regime and to predict the properties of cement. Based on the ƒ-CaO content of clinker, the suitability of the burning conditions was determined, and the optimum conditions of S-0, S-5, S-10, S-15 and S-20 were selected. Figure 2 presents the results of ƒ-CaO content determination in S-0, S-5, S-10, S-15, and S-20. According to Figure 2, the minimum free calcium oxide (ƒ-CaO) contents for S-0, S-5, S-10, S-15, and S-20 are 1.42, 1.31, 1.16, 0.78, and 0.98, respectively, with the corresponding calcination conditions being 1300 °C for 2.5 h, 1300 °C for 2.5 h, 1350 °C for 2.0 h, 1350 °C for 2.0 h, and 1350 °C for 1.5 h.

In S-10, from 1300 °C for 1.5 h to 1300 °C for 2.5 h, due to the increased holding time and liquid phase, more C_2_S combined with CaO to form C_3_S, resulting in a downward trend in ƒ-CaO content. From 1300 °C for 2.5 h to 1350 °C for 1.5 h, although the calcination temperature increased, the significant reduction in holding time led to an increase in ƒ-CaO content. As the calcination temperature and holding time continued to increase, from 1350 °C for 2.0 h to 1350 °C for 2.5 h, the liquid phase decreased, leading to a reduction in ƒ-CaO content. From 1350 °C for 2.0 h to 1350 °C for 2.5 h, due to increased holding time, the clinker became “overburned”, causing a gradual increase in ƒ-CaO content. In S-15, from 1300 °C for 1.5 h to 1300 °C for 2.0 h, with the increase in holding time and liquid phase, more C_2_S combined with CaO to form C_3_S, resulting in a downward trend in ƒ-CaO content. From 1300 °C for 2.0 h to 1300 °C for 2.5 h, although the calcination temperature increased, the significant reduction in holding time led to an increase in ƒ-CaO content. As the calcination temperature and holding time continued to increase, from 1300 °C for 2.5 h to 1350 °C for 2.0 h, due to increased liquid phase, the ƒ-CaO content decreased. From 1350 °C for 2.0 h to 1350 °C for 2.5 h, the increase in holding time led to “overburned” clinker, resulting in a gradual increase in ƒ-CaO content. In S-20, from 1300 °C for 1.5 h to 1300 °C for 2.0 h, due to increased holding time and liquid phase, more C_2_S combined with CaO to form C_3_S, resulting in a downward trend in ƒ-CaO content. From 1300 °C for 2.0 h to 1300 °C for 2.5 h, although the calcination temperature increased, the significant reduction in holding time led to an increase in ƒ-CaO content. As the calcination temperature increased from 1300 °C for 2.5 h to 1350 °C for 1.5 h, the ƒ-CaO content decreased. Finally, from 1350 °C for 1.5 h to 1350 °C for 2.5 h, the increased holding time led to “overburned” clinker, causing the ƒ-CaO content to increase gradually.

The burnability of S-0, S-5, S-10, S-15, and S-20 was analyzed by determining their ƒ-CaO content, the optimal calcination temperature and holding time for S-0, S-5, S-10, S-15, and S-20 were determined as shown in Table 3.

### 3.3. The Effect of Stainless Steel Slag on the Phase Composition of Clinker

**Figure 3:**
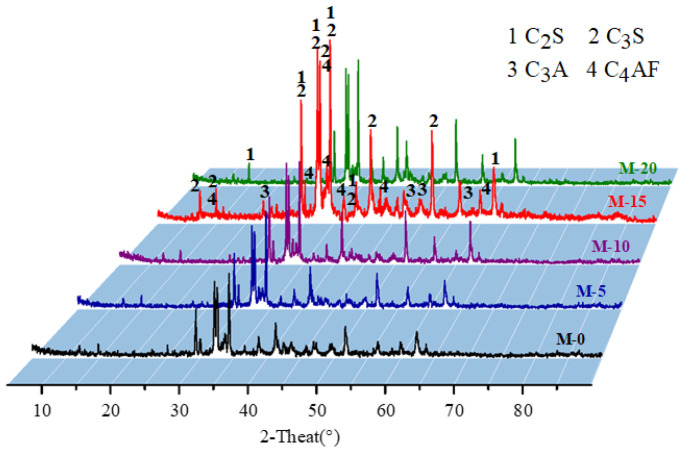
XRD patterns of M-0, M-5, M-10, M-15, and M-20 clinker.

Figure 3 shows the XRD patterns of clinkers M-0, M-5, M-10, M-15, and M-20. Table 6 presents the analysis results of the mineral phases in M-0, M-5, M-10, M-15, and M-20. It can be observed from Figure 1 and Table 6 that the main mineral phases of the five clinkers were C_2_S, C_3_S, C_3_A, and C_4_AF and that the content of free lime in all five clinkers was very low, thus not detectable in the XRD patterns. This indicates that the use of stainless steel slag does not affect the formation of characteristic mineral phases of Portland cement clinker [30]. From M-0 to M-15, the diffraction peak shapes of C_2_S and C_3_S become sharper, and the intensities of the diffraction peaks also increase, indicating better formation of C_2_S and C_3_S. The intensity of the diffraction peak of C_4_AF stabilizes within a certain range before showing a slight increase, which is a result of the introduction of iron elements from stainless steel slag. The changes in the diffraction peak shape of C_3_A are relatively small, and the regularity is not obvious. This indicates that with the increase in stainless steel slag content and the rise in calcination temperature, more liquid phases of C_3_A and C_4_AF appear, facilitating better combination of C_2_S and CaO to form C_3_S. From M-15 to M-20, there is a slight decrease in the intensity of the diffraction peak of C_3_S, while the changes in C_3_A and C_4_AF are minor. It can be observed that from M-0 to M-20, the content of C_3_S gradually increases to the highest level with M-15 before decreasing, while the contents of C_3_A and C_4_AF change slowly and stabilize within a certain range. The variation patterns of C_2_S and C_3_S are evident, with an increase in C_3_S content and a decrease in C_2_S content, indicating a balance between the two. This is because in M-0 to M-20, chemical reactions and solid solution phenomena occur among the oxides CaO, Al_2_O_3_, Fe_2_O_3_, SiO_2_, and SO_3_ in the raw materials due to changes in calcination temperature and holding time, resulting in changes in the properties of the liquid phase and ultimately the mineral phase content.

From M-0 to M-15, as the content of stainless steel slag increases and the calcination temperature rises, the amount of liquid phase gradually increases, accelerating the reaction between C_2_S and CaO. This allows for sufficient time and space for the development of C_3_S, promoting its nucleation and growth. Additionally, the diffraction peak intensities of intermediate phases C_3_A and C_4_AF slightly increase, serving as solvents in the formation process of C_3_S. With the increase in calcination temperature, the amount of liquid phase increases, but the viscosity of the liquid phase decreases, facilitating the growth and development of C_3_S. Moreover, as the content of stainless steel slag increases, the contents of CaO, SiO_2_, Al_2_O_3_, and Fe_2_O_3_ also increase, leading to a sufficient reaction from C_2_S to abundant C_3_S. From M-15 to M-20, the decrease in C_3_S content is due to the increase in stainless steel slag content, resulting in an increase in Fe content. The decrease in holding time reduces the amount of liquid phase in the environment, causing CaO to react insufficiently, partly existing in solid phase, resulting in low C_3_S content and fine grains. The increase in C_4_AF content is attributed to the decrease in holding time, leading to an increase in intermediate phase and a reduction in melting liquid phase, resulting in a decrease in C_3_S content and hindering the nucleation and development of C_3_S crystals. Therefore, from M-0 to M-15, the diffraction peak of the C_3_S phase gradually increases, while from M-15 to M-20, the diffraction peak of the C_3_S phase decreases. Thus, the XRD pattern of M-15 represents the optimal composition with C_3_S, C_2_S, C_3_A, and C_4_AF phases.

### 3.4. The Impact of Stainless Steel Slag on the Microstructure of Clinker

**Figure 4:**
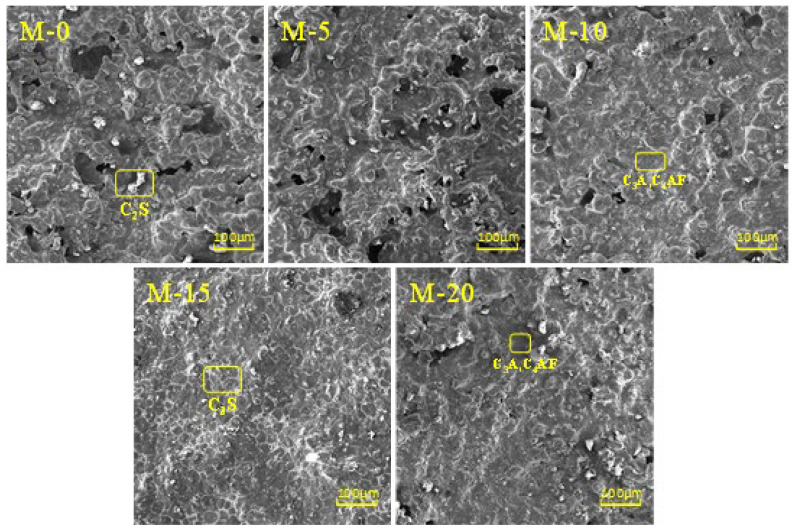
Typical SEM morphology of M-0, M-5, M-10, M-15, and M-20.

The microstructure of clinker directly influences the physical properties of Portland cement clinker. Scanning electron microscopy (SEM) provides a straightforward method to observe the microstructure of clinker. Figure 4 shows the typical SEM micrographs of M-0, M-5, M-10, M-15, and M-20. In these micrographs, C_3_S minerals appear as hexagonal plates, while C_2_S minerals appear as circular shapes. The intermediate phases C_3_A and C_4_AF, depicted in black and light gray, respectively, exhibit dendritic structures [31]. From Figure 4, it is evident that well-crystallized minerals are C_2_S and C_3_S crystals. From M-0 to M-15, the microstructure of clinker gradually becomes smoother, and the surface irregularities decrease. In M-0, M-5, and M-10, numerous prismatic and blocky particles are present, distributed evenly with clear edges, which are identified as C_3_S crystals. Circular and irregularly shaped particles, identified as C_2_S crystals, are also observed, with larger sizes. The intermediate phases among C_2_S crystals are C_3_A and C_4_AF crystals, mainly tubular in shape with clear boundaries and numerous pores. Unreacted ƒ-CaO is also observed. In M-15, the minerals are more evenly distributed, with a large number of continuous blocks identified as C_3_S crystals. These crystals are well developed, hexagonal in shape, with clear boundaries and large sizes. C_2_S appears as spherical grains, smaller in size, while the intermediate phases of C_3_A and C_4_AF are already melted and sintered together, forming a dense structure distributed around the C_3_S crystals. In M-20, a large number of C_2_S and C_3_S crystals are generated, with clear boundaries and larger sizes for the C_2_S crystals. The intermediate phases of C_3_A and C_4_AF are already melted and sintered together, distributed around the C_2_S and C_3_S crystals. Overall, from M-0 to M-15, an increase in stainless steel slag content enhances the aggregation of C_3_S crystals, resulting in more regular continuous distribution and significant mineral blockage. From M-15 to M-20, there is a slight dispersal of C_3_S crystals, with a decrease in liquid-phase content. These results indicate that stainless steel slag promotes the generation of C_3_S crystals in clinker, and M-15 exhibits favorable crystal structures and contents of C_2_S, C_3_S, C_3_A, and C_4_AF, consistently with the XRD analysis results.

**Figure 5:**
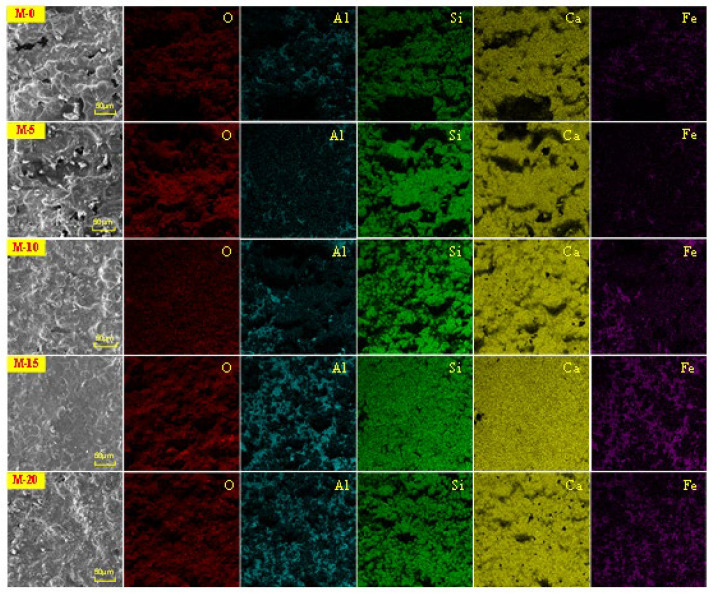
Analysis results of M-0, M-5, M-10, M-15, and M-20 element surfaces.

Figure 5 shows the elemental surface analysis results of M-0, M-5, M-10, M-15, and M-20. From the figure, it can be observed that in the five types of clinker, there is good overlap among the elements Ca and Si; Al and Ca; and Al, Fe, and Ca. In M-0 and M-5, the distribution of Al and Fe elements is relatively dispersed and scattered within the Ca and Si elements, indicating higher content of Ca and Si in M-0 and M-5, with Al and Fe distributed around the Ca and Si phases. In M-10, the distribution of Ca and Si elements becomes more uniform, while the distribution of Al and Fe elements becomes more localized, mainly concentrated in the lower-left area. This suggests that with the increase in stainless steel slag content, there is a slight increase in the content of C_3_A and C_4_A phases generated by Al, Fe, and Ca, which are melted and sintered together. In M-15, the Ca and Si elements are more abundant and evenly distributed, while the Al and Fe elements show a significant increase in content and are widely distributed in regions. This indicates that M-15 has optimal Ca and Si phases, as well as Al and Ca phases and Al, Fe, and Ca phases, with the Al, Fe, and Ca phases being distributed in the gaps around the Ca and Si phases. In M-20, the distribution of Ca, Si, Al, and Fe elements becomes more localized, with little change in the content of Al and Fe elements and a slight decrease in the content of Ca and Si elements. These observations are consistent with the XRD analysis results.

From M-0 to M-20, the contents of O, Ca, and Si elements all initially increase until M-15 and then decrease, while the contents of Al and Fe elements gradually increase and stabilize in M-15 and M-20.

**Figure 6:**
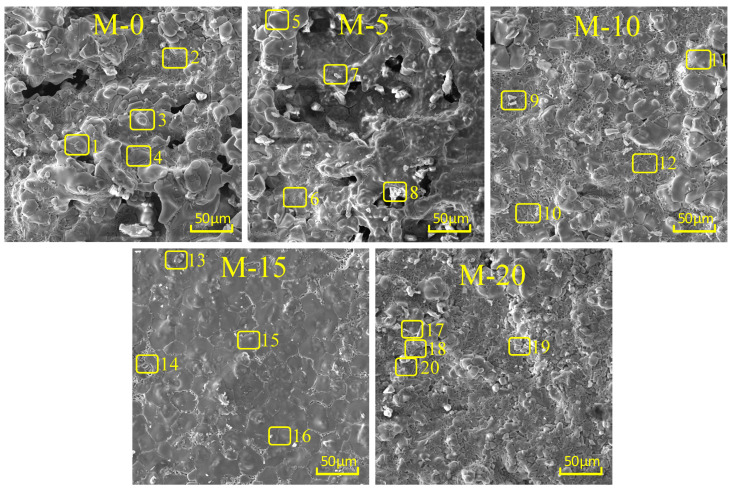
Analysis of M-0, M-5, M-10, M-15, and M-20 energy spectrum points (1–20).

Figure 6 shows the energy spectrum point analysis for M-0, M-5, M-10, M-15, and M-20, while Table 7 presents the corresponding results. It is observed from Table 7 that all five types of clinkers (M-0, M-5, M-10, M-15, and M-20) contained four minerals: C_2_S, C_3_S, C_3_A, and C_4_AF. Based on the energy spectrum point analysis, specific microstructures of these minerals were identified. In Figure 6, points 4, 5, 10, 15, 16, and 17 represent C_3_S minerals; points 1, 3, 4, 8, 9, 13, and 19 represent C_2_S minerals; and points 1, 2, 6, 8, 10, 12, 14, 18, and 20 correspond to intermediate phases of C_3_A and C_4_AF. C_3_S crystals contain minimal Si solid solution but incorporate a certain amount of Al and a small amount of Fe. Both C_2_S and intermediate phases contain a certain amount of Si solid solution, with the Si content in the intermediate phases being slightly higher than that in C_2_S. It is evident from Figure 6 that at 1000× magnification in scanning electron microscopy, various crystal minerals in clinker can be clearly observed, with C_3_S crystals appearing hexagonal, C_2_S crystals appearing circular, and intermediate phases of C_3_A and C_4_AF appearing dendritic. For M-0, M-5, and M-10, points 1, 4, 7, and 9 indicate the presence of numerous C_2_S minerals, mostly distributed as granular and irregular particles in concentrated positions. In M-15, points 15 and 16 reveal the abundance of C_3_S minerals, mostly in the form of continuous blocks with uniform distribution and distinct edges. In M-20, points 18 and 20 show that most of the intermediate phases of C_3_A and C_4_AF have fused together, forming a dense structure mainly in tubular shape with clear boundaries and numerous pores. Consequently, in M-15, C_3_S minerals are more continuously distributed with a denser structure, with a small amount of C_2_S minerals attached to C_3_S minerals, and the intermediate phases of C_3_A and C_4_AF are distributed continuously around the gaps of C_3_S minerals, which is consistent with the typical morphology and elemental surface analysis results of M-0, M-5, M-10, M-15, and M-20.

### 3.5. The Effect of Stainless Steel Slag on the Compressive Strength of Cement

In the compressive strength test for Portland cement, 3 days, 7 days, and 28 days are typically chosen to measure its compressive strength. C_2_S has relatively low early strength but develops well over time; C_3_A releases a lot of heat and sets and hardens quickly, resulting in high early strength but no further strength growth in the later stages; the iron-phase solid solution in cement, which is close to C_4_AF in Portland cement clinker, has an early hydration rate between those of C_3_A and C_3_S, providing early strength similar to C_3_A and later strength more like C_2_S. This phase also has good impact resistance and sulfate attack resistance. Thus, the 3 days, 7 days, and 28 days compressive strength tests were chosen for evaluating Portland cement.

**Figure 7:**
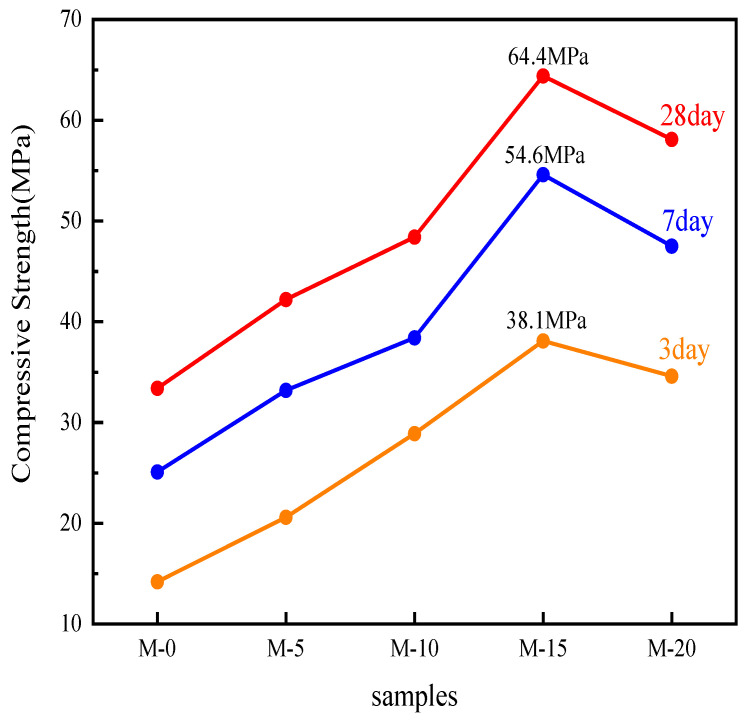
Compressive strength of cement at different detection cycle ages.

Figure 7 shows the compressive strength of M-0, M-5, M-10, M-15, and M-20 at 3 days, 7 days, and 28 days, respectively. It can be observed that the compressive strength of M-0, M-5, M-10, M-15, and M-20 gradually increased at 3 days, 7 days, and 28 days, indicating that all five types of clinkers underwent hydration reaction. In the detection cycle of 3 days, 7 days, and 28 days, the compressive strength of M-15 reached the maximum values, which were 38.1 MPa, 54.6 MPa, and 64.4 MPa, respectively. Cement hydration mainly occurs within the first seven days, during which more than 90% of the strength is developed. The porosity of stainless steel slag was greater than that of ordinary Portland cement. This resulted in Portland cement clinker calcined from stainless steel slag having larger pores, providing more space for cement hydration. Additionally, since M-15 and M-20 contained higher amounts of C_2_S and C_3_S, they generated more C-S-H, leading to more thorough hydration reactions. As a result, M-15 and M-20 exhibited higher compressive strength in the detection cycle compared with M-0, M-5, and M-10.

At 3 days, 7 days, and 28 days, the compressive strength of M-0, M-5, M-10, and M-15 showed a gradual increasing trend, while that of M-15 and M-20 showed a decreasing trend. This is because in M-0, M-5, M-10, and M-15, the increasing sintering temperature led to more liquid phases of C_3_A and C_4_AF, facilitating the better combination of C_2_S and CaO to generate C_3_S, promoting the formation of C_2_S and C_3_S, and inducing hydration reactions to produce more C-S-H, thus benefiting the early compressive strength of cement. The final growth of more and larger quantities of ettringite crystals in the cement hardening body is advantageous for increasing the compressive strength of cement, and the higher stainless steel slag content will form more ettringite, benefiting the compressive strength of cement. Moreover, the slight increase in C_3_A from M-0 to M-15 avoided excessive expansion caused by too much C_3_A in the later stage. Consequently, the compressive strength gradually increased from M-0 to M-15 at various ages. C_3_S, as the most important mineral phase of cement clinker, guarantees the hardening of cement. A decrease in its content will lead to a decrease in the compressive strength of cement. From M-15 to M-20, the decrease in sintering time was unfavorable for the formation of C_2_S to C_3_S, resulting in a gradual decrease in the content of C_3_S in M-15 and M-20. Therefore, the compressive strength gradually decreased from M-15 to M-20 at various ages. Thus, it can be concluded that with the increase in stainless steel slag content, the compressive strength of Portland cement gradually increases at various detection cycle ages. When the stainless steel slag content was 15%, the compressive strength reached its maximum value, and as the stainless steel slag content continued to increase, the compressive strength gradually decreased.

### 3.6. The Influence of Stainless Steel Slag on the Free Lime Content in Cement Clinker

The standard “Cement Clinker for Ordinary Portland Cement” (GB/T 21372-2008) [32] explicitly specifies the limit for free lime content in clinker, stating that ƒ-CaO content in ordinary Portland cement clinker shall not exceed 1.5%. If ƒ-CaO content in clinker exceeds 1.5%, it will lead to a 1.98-fold volume expansion in Ca(OH)_2_ during the hydration of cement, resulting in inadequate strength of cement. Conversely, if ƒ-CaO content in clinker is less than 0.5%, clinker tends to be overburned, resulting in poor reactivity and low strength.

**Figure 8:**
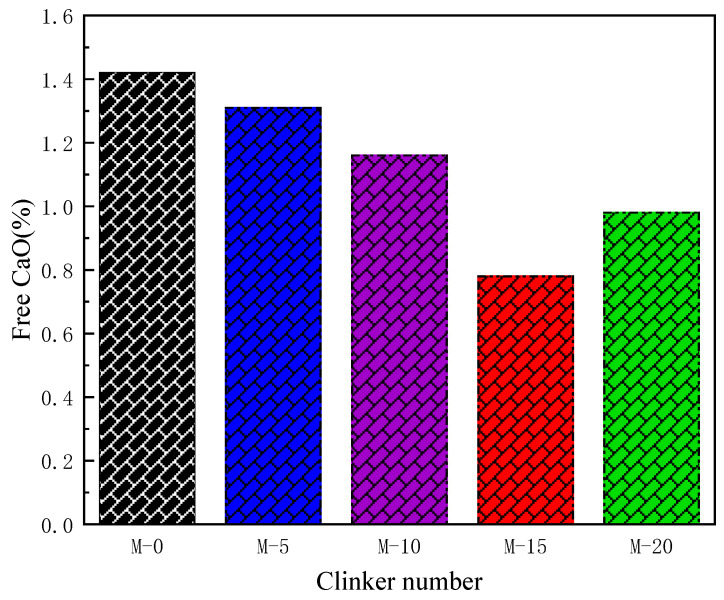
Free calcium oxide content in M-0, M-5, M-10, M-15, M-20.

Figure 8 illustrates the determination results of ƒ-CaO content in M-0, M-5, M-10, M-15, and M-20. It can be observed from the figure that ƒ-CaO contents in the corresponding clinker samples of M-0, M-5, M-10, M-15, and M-20 were all less than 1.5%, indicating that under the given calcination conditions, the development of silicate minerals in clinker was sufficient. Moreover, ƒ-CaO content gradually decreased from M-0 to M-15, reaching the lowest value of 0.78% at M-15. This is attributed to the increase in the contents of SiO_2_, Al_2_O_3_, and Fe_2_O_3_ from M-0 to M-15 due to the addition of stainless steel slag. As a result, more CaO combined with these components. Additionally, the increase in calcination temperature and time promoted the formation of clinker, leading to a gradual decrease in ƒ-CaO content. However, from M-15 to M-20, due to the further increase in iron and silicon phases, some C_3_S remained incompletely reacted during calcination, resulting in secondary ƒ-CaO. Furthermore, the reduction in holding time led to a decrease in the amount of liquid phase in the environment, causing some CaO to exist in the solid phase. This led to an increase in ƒ-CaO content and porosity, resulting in a decrease in C_3_S content and finer grain size. Therefore, in M-20, there was an increase in ƒ-CaO content. It can be concluded that in M-15, the ƒ-CaO content in clinker was the lowest, indicating higher reactivity and favoring the formation of C_3_S.

## 4. Conclusions

This study investigated Portland cement clinker burned with different contents of stainless steel slag (S-0, S-5, S-10, S-15, and S-20), and the following conclusions were drawn.

(1) Through analysis of the chemical composition and mineral phases of stainless steel slag, it was found that stainless steel slag contains both calcium oxide present in Portland cement raw materials and minerals such as C_2_S and C_3_S found in Portland cement clinker. Therefore, stainless steel slag can be utilized as an independent raw-material component for burning Portland cement clinker.

(2) The optimal burning temperature and holding time for S-0, S-5, S-10, S-15, and S-20 are 1300 °C for 2.5 h, 1300 °C for 2.5 h, 1350 °C for 2.0 h, 1350 °C for 2.0 h, and 1350 °C for 1.5 h, respectively. Utilizing stainless steel slag for burning Portland cement clinker can reduce the burning temperature and holding time, thereby lowering the cost and energy consumption of industrial production and achieving carbon emission reduction goals.

(3) With the increase in stainless steel slag content, the C_3_S phase of Portland cement clinker gradually increases first and then decreases, while the C_3_A and C_4_AF phases gradually and slowly increase. The C_3_S crystals transition from granular to continuous honeycomb-like distribution, while the intermediate phases of C_3_A and C_4_AF crystals gradually melt and sinter together, leading to their distribution around the C_2_S and C_3_S crystals. ƒ-CaO content shows a trend of decreasing first and then increasing. When the stainless steel slag content is 15%, the Portland cement clinker has the highest C_3_S phase content. The C_3_S crystals are predominantly large continuous hexagonal plates with well-developed structures and relatively dense formation, while the C_2_S crystals are spherical and smaller in size. The intermediate phases of C_3_A and C_4_AF crystals melt and sinter together, leading to their distribution in the gaps around the C_3_S crystals. The ƒ-CaO content is at its lowest, at 0.78%.

(4) With the increase in stainless steel slag content, the compressive strength of Portland cement at various curing ages first increases gradually and then decreases, yet all can meet the requirements of the national standard for ordinary Portland cement. When stainless steel slag is added up to 15%, the compressive strength at a curing age of 28 days reaches its maximum value of 64.4 MPa, allowing for the preparation of high-strength clinker such as P.O 62.5. This meets the requirements for producing cement of grades P.O 42.5 and P.O 52.5 (or even P.O 62.5), suggesting the potential of utilizing stainless steel slag as a substitute raw material in the cement industry. This contributes to increasing the comprehensive utilization rate of stainless steel slag and achieving the goal of clean production of Portland cement clinker.

## Figures and Tables

**Table 1:** Phase proportions of stainless steel slag (mass fraction/%).

	Ca_2_SiO_4_	CaO	Ca_3_SiO_5_	CaCr_2_O_4_	Ca(OH)_2_	Ca_4_Si_2_O_7_F_2_
Stainless steel slag	48.0	2.00	7.77	0.31	4.01	2.78
	**MgO**	**MgCO_3_**	**Fe_2_O_3_**	**(Fe,Mg)(Cr,Fe)_2_O_4_**	**Ca_2_Al_2_SiO_7_**	**Ca_3_MgSi_2_O_8_**
Stainless steel slag	0.81	3.69	5.27	8.92	2.61	3.31

**Table 2:** Calcination scheme for Portland cement clinker.

Calcination Temperature	Holding Time
1300 °C	1.5 h
	2.0 h
	2.5 h
1350 °C	1.5 h
	2.0 h
	2.5 h

**Table 3:** Optimal calcination conditions for S-0, S-5, S-10, S-15, and S-20.

Samples	S-0	S-5	S-10	S-15	S-20
Optimal burning conditions	1300 °C-2.5 h	1300 °C-2.5 h	1350 °C-2.0 h	1350 °C-2.0 h	1350 °C-1.5 h

**Table 4:** Oxide composition table of raw materials (%).

Composition	SiO_2_	Al_2_O_3_	Fe_2_O_3_	CaO	MgO	TiO_2_
Stainless steel slag	25.78	3.02	3.58	43.70	7.88	0.65
Silica rock	94.42	2.70	0.32	0.22	0.13	0.09
Limestone	2.37	1.56	0.44	52.27	1.05	0.04
Copper smelting slag	31.96	3.62	57.89	1.81	1.10	0.32
Basalt	56.74	14.84	7.45	4.09	4.40	0.69
**Composition**	**Na_2_O**	**MnO**	**BaO**	**P_2_O_5_**	**SO_3_**	**Total**
Stainless steel slag	0.17	0.63	0.05	0.14	0.45	99.68
Silica rock	0.32	0.00	0.03	0.05	0.05	99.86
Limestone	0.14	0.00	0.00	0.02	0.08	99.87
Copper smelting slag	0.40	0.06	0.07	0.06	0.28	97.57
Basalt	1.69	0.11	0.12	0.15	0.27	99.95

**Table 5:** Raw-material ratio of calcined Portland cement clinker (mass percentage%).

Sample	Stainless Steel Slag	Silica Rock	Limestone	Copper Smelting Slag	Basalt
S-0	0.00	4.63	80.43	1.62	13.31
S-5	5.02	3.39	76.72	1.44	13.34
S-10	10.00	2.20	73.01	1.25	13.53
S-15	15.00	1.93	69.69	1.10	12.28
S-20	20.08	0.70	65.90	1.14	12.18

**Table 6:** Mineral composition of M-0, M-5, M-10, M-15, and M-20 (%).

Samples	C_3_S	C_2_S	C_3_A	C_4_AF
M-0	40.76	45.47	6.82	6.93
M-5	42.24	42.91	7.81	7.04
M-10	46.54	34.31	10.81	8.32
M-15	54.62	25.21	9.12	11.03
M-20	48.98	27.92	11.22	11.86

**Table 7:** Results of M-0, M-5, M-10, M-15, and M-20 energy spectrum point analysis.

Clinker	Location	O	Al	Si	Ca	Fe	Mineral Phase Types
M-0	1	24.88	6.05	4.82	21.76	2.49	C_3_S + C_3_A
	2	22.11	1.15	1.64	56.48	18.62	C_4_AF
	3	64.10	1.85	10.00	24.85	1.20	C_2_S + ƒ-CaO
	4	67.21	0.72	8.66	23.24	0.18	C_2_S
M-5	5	51.37	0.65	11.08	36.48	0.41	C_3_S
	6	59.52	8.72	4.31	24.88	2.57	C_3_A + C_4_AF
	7	73.54	0.27	9.24	16.80	0.15	C_2_S
	8	71.07	1.54	7.07	19.87	0.44	C_2_S + C_3_A
M-10	9	59.11	0.89	12.14	27.74	0.12	C_2_S
	10	58.93	8.25	4.05	26.55	2.22	C_3_S + C_2_S
	11	62.26	0.79	9.44	27.24	0.26	C_3_S
	12	53.55	11.68	2.81	26.53	5.43	C_3_A + C_4_AF
M-15	13	70.68	1.21	8.72	18.94	0.45	C_2_S
	14	66.05	9.67	2.54	18.17	4.56	C_3_A + C_4_AF
	15	40.67	0.71	14.49	43.60	0.53	C_3_S
	16	49.35	0.88	12.37	36.93	0.48	C_3_S
M-20	17	59.17	0.95	9.64	30.05	0.20	C_3_S
	18	21.01	2.05	1.73	60.32	14.89	C_4_AF
	19	65.10	2.12	9.56	22.00	1.22	C_2_S
	20	42.06	20.83	4.90	30.17	2.05	C_3_A

## Data Availability

The original contributions presented in the study are included in the article, further inquiries can be directed to the corresponding author/s.
